# Global Sleep Quality and Uncontrolled Eating Among Romanian Healthcare Students: The Mediating Pathway of Emotional Eating

**DOI:** 10.3390/clockssleep8030057

**Published:** 2026-09-21

**Authors:** Iustina-Gabriela Mihăianu, Andreea-Sabina Butucaru, Magdalena Iorga, Raluca Ortensia Cristina Iurcov

**Affiliations:** 1 Faculty of Medicine, ”Grigore T. Popa” University of Medicine and Pharmacy, 700115 Iasi, Romania; nutr-rom-514@students.umfiasi.ro (I.-G.M.); nutr-rom-487@students.umfiasi.ro (A.-S.B.); 2 Faculty of Psychology and Educational Sciences, “Alexandru Ioan Cuza” University of Iasi, 700554 Iasi, Romania; 3 Faculty of Medicine and Pharmacy, University of Oradea, 410087 Bihor, Romania; riurcov@uoradea.ro

**Keywords:** emotional eating, cognitive restraint, uncontrolled eating, sleep quality, university students, late night eating, diet, health

## Abstract

Background: Healthcare university students face disruptive sleep schedules and irregular meals caused by busy schedules, multiple activities, and stressful internships. This study aimed to investigate the complex relationships between sleep quality, psychological eating dimensions (cognitive restraint, uncontrolled eating, and emotional eating), accommodation, history of diets and late-night food intake, while evaluating the impact of demographic factors such as age, sex, and residential background. Methods: A cross-sectional survey was conducted among a sample of Romanian healthcare university students (*n* = 265; 74.3% women). Psychometric evaluation included the Pittsburgh Sleep Quality Index (PSQI) and the Three-Factor Eating Questionnaire (TFEQ-R21). Sociodemographic data (age, gender, environment and accommodation) and information about diets were collected. Late-night food intake was defined as eating after 21:00. Data processing and analysis were performed using IBM Statistical Package for the Social Sciences (SPSS) for Windows, version 26 (SPSS Inc., Chicago, IL, USA). Results: Students exhibit poor global sleep quality, which positively correlated with all TFEQ-R21 subscales. Students with a prior history of following weight-loss diets demonstrated significantly elevated levels of intentional food monitoring and psychological eating disinhibition compared to non-dieters. Urban students feel greater psychological pressure to constantly and consciously control what they eat. Students living in dormitories (Mdn = 2.50, IQR = 1.83) and those living in rented apartments (Mdn = 2.17, IQR = 1.50) reported significantly higher Emotional Eating scores compared to students residing with their parents. Male students demonstrated a substantially higher prevalence of late-night food consumption compared to female students (77.9% vs. 59.4%). Cognitive restraint and female gender protect against nighttime snacking, while uncontrolled eating and poor-quality sleep significantly increase this risk. After adjusting for age, sleep disturbances were positively associated with emotional eating (Path a: B = 0.0967, β = 0.320, *p* < 0.001, R^2^ = 0.109), which in turn was strongly associated with uncontrolled eating (Path b: B = 0.4621, β = 0.709, *p* < 0.001). While the model explained 52.16% of the variance in uncontrolled eating, this was driven almost entirely by path b, as the initial contribution of sleep was considerably smaller (Path a: R^2^ = 0.109), yielding a significant standardized indirect association (β = 0.227, 95% BootCI [0.148, 0.309]). Conclusions: Sleep disturbances are linked to uncontrolled eating patterns primarily through their shared indirect association with emotion-driven eating. Prioritizing weight loss over health promotion increases students’ psychological vulnerability.

## 1. Introduction

The transition to higher education represents a critical developmental period frequently characterized by profound lifestyle disruptions, academic stress, and irregular schedules. Within healthcare education, students face a distinctive combination of chronic stressors, including demanding academic curricula, intensive examination periods, clinical shift rotations, and early exposure to patients’ suffering. Although these challenges are commonly considered integral to professional training, increasing evidence indicates that they may substantially compromise student well-being. Their impact may be further intensified by everyday responsibilities associated with the transition to independent living, including financial autonomy, managing living conditions, maintaining relationships with peers, living in large cities and preparing meals. Together, these stressors may contribute to sleep disturbances and disruptions in lifestyle behaviors.

Sleep quality has become an important concern among university students, as insufficient or disrupted sleep is closely associated with psychological distress, impaired self-regulation, and unhealthy eating behaviors. In the general population, sleep problems and poor sleep quality are estimated to affect between 10–48% [1], and diagnosed sleep disorders have been reported in the literature at rates ranging from 5% to 15% [2,3].

Insufficient and poor-quality sleep is also widespread among university students. In Romania, students reported an average sleep duration of 6.71 h per night [4], while in Saudi Arabia, 44% of students were dissatisfied with their sleep quality [5]. In Poland, 36.8% of participants presented symptoms of insomnia [6], whereas in Australia, approximately 78% reported poor sleep quality, which was associated with unhealthy dietary habits, alcohol consumption, and tobacco use [7]. In Hungary, Al-Jawarneh et al. [8] identified that consuming heavy evening meals, substituting snacks for main meals, and having a short meal-to-bedtime interval were significantly associated with poor sleep quality. Sleep quality was impaired in 42.8%, and 17.9% showed clinically relevant scores among university students in Germany and Luxembourg [9].

This issue may be particularly relevant for healthcare students, who are exposed to rigorous academic demands, clinical rotations, heavy coursework, irregular schedules, and frequent stressors related to patient care. In this context, chronic sleep deprivation may interfere with cognitive and metabolic regulation and may contribute to maladaptive dietary patterns, including emotional and uncontrolled eating. Academic schedules and living away from their families increase the risk of adopting unhealthy eating habits. These habits include skipping breakfast, late-night snacking, irregular meal timing, and substituting snacks for a main meal [5]. Therefore, examining the mechanisms that link sleep problems with eating behavior in healthcare students is essential for understanding potential risks to their physical and mental health.

The association between sleep and dietary and lifestyle habits and their sleep quality becomes a public health problem, and researchers focus on emotional eating as an important psychological mechanism reflecting the tendency to consume food in response to negative emotional states such as anxiety, stress or burnout, specific to healthcare students’ lives. According to Grajek et al. [10], emotional eating was found to be characteristic of 37.9% of Polish university students, while Lotfi et al. [11] found a significant relationship between poor sleep quality and emotional, restrained and external eating behaviors among Iranian medical students. Priede et al. [12] also found a strong relationship between emotional eating and sleep-related problems accompanied by stress, anxiety and depression among Latvian healthcare students.

Data highlighted by scientific literature offered diverse perspectives on healthcare students’ lifestyle. For example, studies conducted among healthcare students in the U.S. reported that, compared with peers of the same age, participants demonstrated better health and fitness, which may be partly explained by their greater knowledge of health and nutrition [13]. Some other researchers indicated that healthcare students had lower rates of chronic disease and mortality than other university students [14,15]. But despite this generally favorable health profile, medical students encounter a range of distinctive challenges that may negatively affect their overall well-being. In response to academic pressure and excessive workload, they may reduce their engagement in physical activity, develop unhealthy sleep patterns, and experience shifts in dietary behavior, including irregular meal timing and increased consumption of readily available, high-calorie, and highly palatable foods. Scientific evidence indicates that sleep disturbances may weaken cognitive control and increase stress reactivity, thereby promoting greater reliance on emotion-driven food consumption, including late-night eating and junk food [16,17,18]. Also, Chourdakis et al. [19] identified a strong association between late-night eating and obesity among medical students in Greece. Similarly, Kara et al. [20] reported that examination periods may intensify problematic eating behaviors and contribute to weight gain among students. Giannopoulou et al. [21] reported a high prevalence of eating disorders among university students enrolled in health-related disciplines. Several contributing factors were identified, including relocation away from home, the formation of new social networks, changes in the eating environment, high academic pressure, and the specific demands of academic training. The authors also noted a particularly high prevalence of binge eating, estimated at 41%.

A growing body of research has examined the complex interactions between eating dimensions and sleep quality. But the relationship between these variables needs to be better explained, including the complexity of other sociodemographic factors. The present study aimed to investigate the complex relationships between sleep quality, psychological eating dimensions (cognitive restraint, uncontrolled eating, and emotional eating), accommodation, history of diets and late-night food intake, while evaluating the impact of demographic factors such as age, sex, and residential background. This paper is part of a larger study focusing on eating behaviors among healthcare students, and the hypotheses of the present research are:

**H1.**  *Higher levels of sleep disturbances (indicated by higher PSQI Global Scores) will be positively associated with increased levels of Cognitive restraint, Emotional eating and Uncontrolled eating.*

**H2.**  *Students with a prior history of weight-loss dieting will demonstrate significantly higher levels of Cognitive Restraint, Uncontrolled Eating, and Emotional Eating compared to non-dieters.*

**H3.**  *Female healthcare students will have significantly lower odds of reporting late-night food intake compared to male students.*

**H4.**  *Poorer sleep quality (indicated by higher Global PSQI Scores) will significantly increase the odds of late-night food intake, independent of dietary behaviors.*

## 2. Results

### 2.1. Socio-Demographic Data

The study included a sample of *n* = 265 students, with a mean age of M = 22.00 (SD = 4.46 years, ranging from 18 to 47 years), enrolled in all years of study. The majority of the respondents were female (74.3%), while male students accounted for 25.7%. In terms of residency, 61.9% of the participants originated from urban areas, and 38.1% resided in rural environments.

Regarding housing status during studies, more than half of the respondents lived in rented apartments (57.0%), 22.3% resided in university dormitories, and 20.8% lived with their parents.

More than half of the respondents, 170 (64.2%), declared that they eat late-night meals (after 9:00 PM), and 99 (37.4%) of them have a history of weight-loss diets.

### 2.2. Three-Factor Eating Questionnaire (TFEQ-R21)

The psychological dimensions of eating behavior were captured utilizing the revised 21-item Three-Factor Eating Questionnaire (TFEQ-R21). The instrument treats its three structural dimensions as independent continuous subscales, standardly calculated on a 1.00–5.00 mean scale. Comprehensive psychometric parameters and descriptive indices for all TFEQ-R21 dimensions are summarized in Table 1.

**Figure 1:**
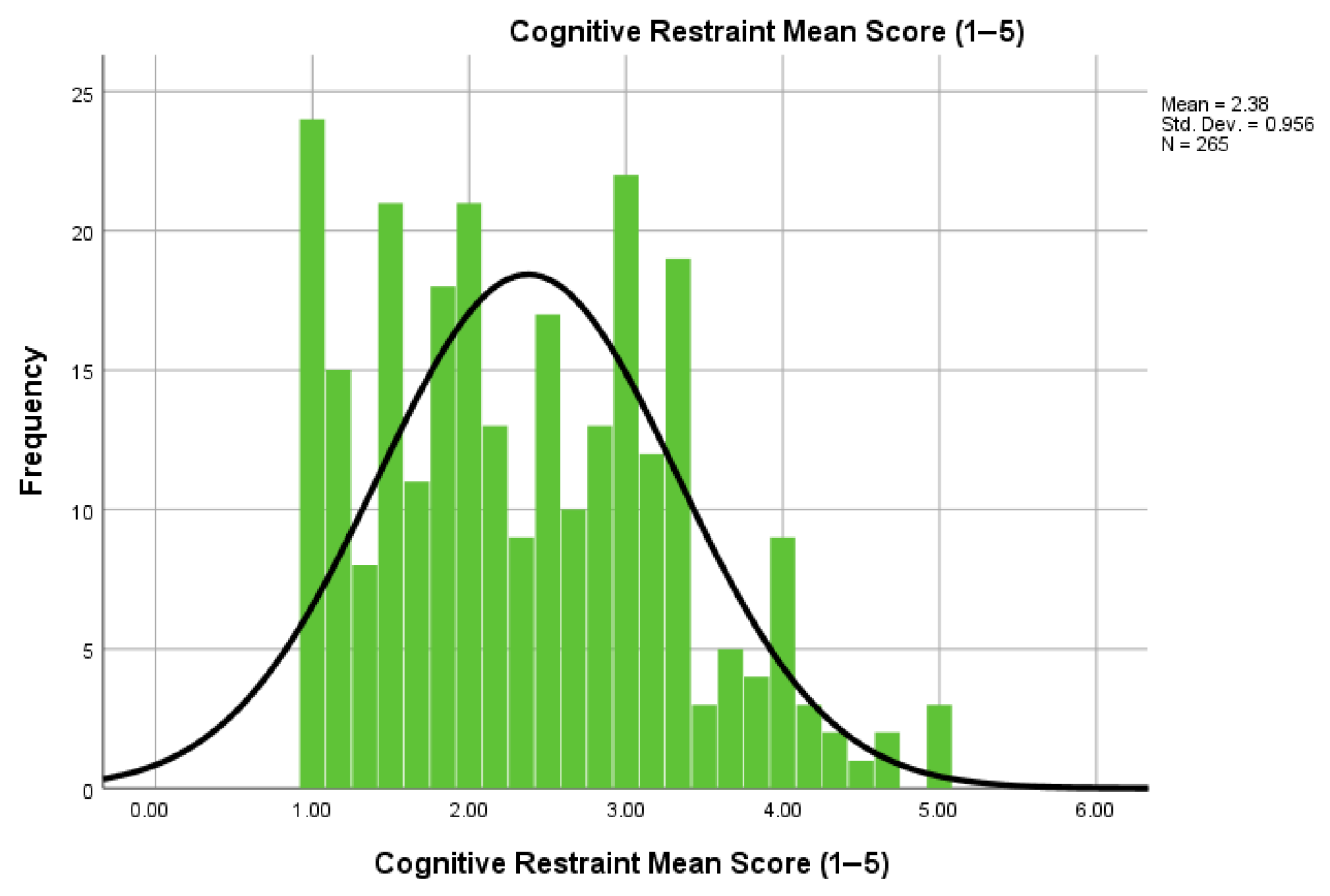
Distribution of the standardized TFEQ-R21 Cognitive Restraint score (*n* = 265, M = 2.38 ± 0.956). The continuous black curve represents the theoretical normal distribution curve.

The evaluation of conscious dietary monitoring via the Cognitive Restraint subscale demonstrated excellent internal consistency for its 6-item structure (α = 0.910). At the sample level (*n* = 265), the mean score was M = 2.38 (SD = 0.956). The categorical distribution revealed that 52.8% (*n* = 140) of participants presented low cognitive restraint, 38.1% (*n* = 101) exhibited moderate restraint, and 9.1% (*n* = 24) reported high intentional food restriction (see Figure 1).

**Figure 2:**
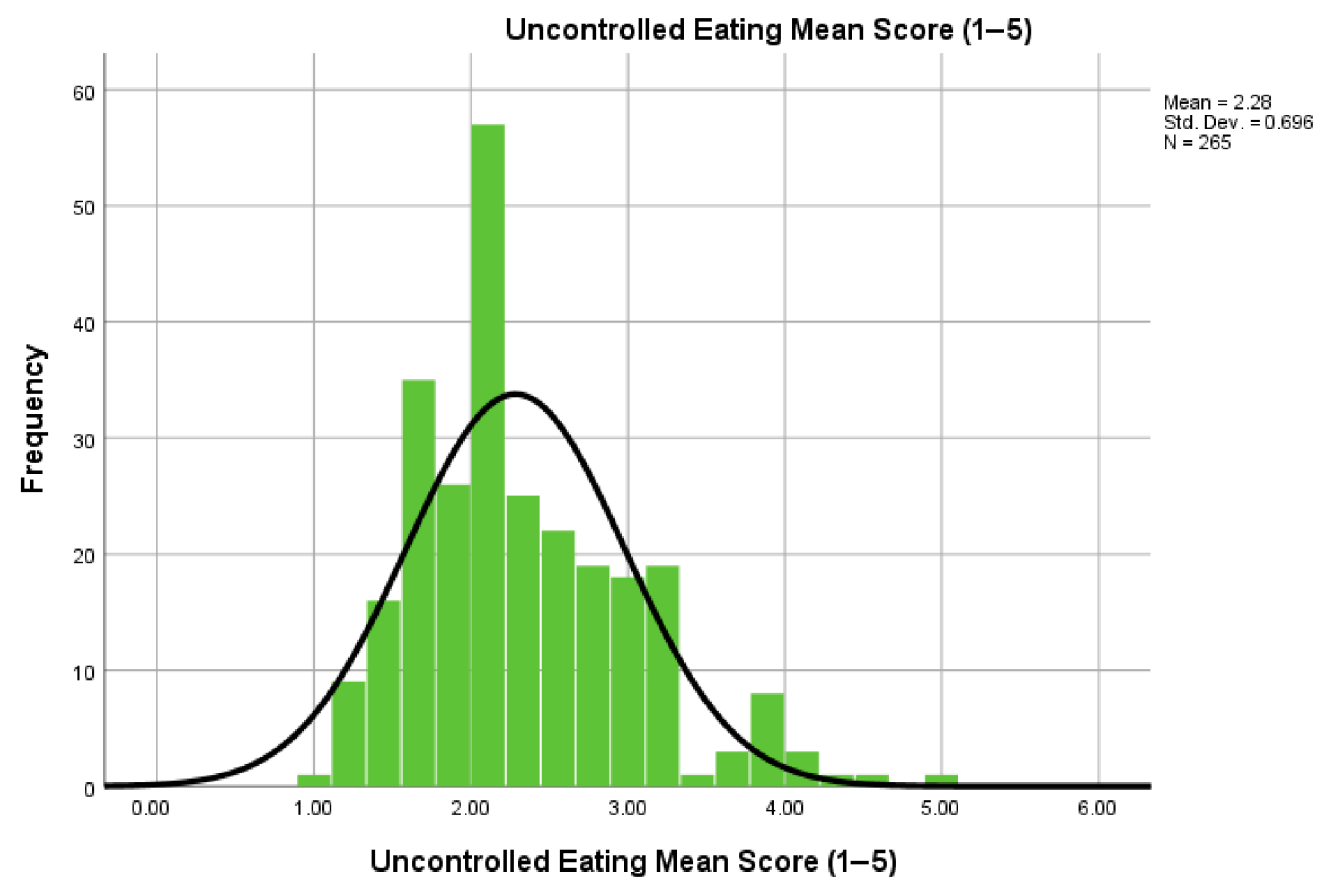
Distribution of the standardized TFEQ-R21 Uncontrolled Eating score (*n* = 265, M = 2.28 ± 0.695). The continuous black curve represents the theoretical normal distribution curve.

The tendency to overeat in response to external food cues or a loss of satiety control was operationalized through the Uncontrolled Eating subscale. Internal reliability for this 9-item dimension was robust (α = 0.879). The subscale displayed a sample mean of M = 2.28 (SD = 0.695). Overall, 63.8% (*n* = 169) of respondents were classified in the low manifestation bracket, 30.9% (*n* = 82) presented moderate uncontrolled eating, and 5.3% (*n* = 14) demonstrated high dietary disinhibition (see Figure 2).

**Figure 3:**
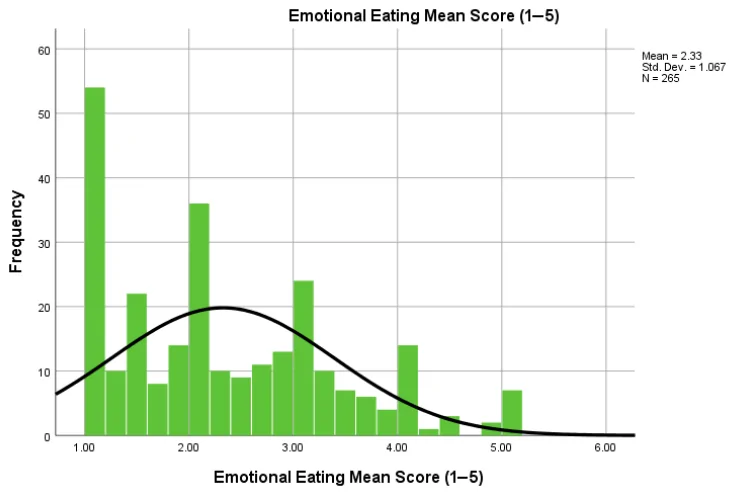
Distribution of the standardized TFEQ-R21 Emotional Eating score (*n* = 263, M = 2.33 ± 1.067). Two participants were excluded from this subscale analysis due to incomplete response profiles (n = 265 total sample). The continuous black curve represents the theoretical normal distribution curve.

Lastly, the Emotional Eating subscale quantified food consumption triggered by negative affective states. The 6-item construct exhibited outstanding internal reliability (α = 0.948). Descriptive profiling indicated a sample mean of M = 2.33 (SD = 1.067). Distribution across these descriptive manifestation tiers showed that 58.1% (*n* = 154) of students exhibited low emotional eating, 30.2% (*n* = 80) fell into the moderate category, and 11.7% (*n* = 31) displayed high affect-driven eating behavior (see Figure 3).

### 2.3. Pittsburgh Sleep Quality Index (PSQI)

At the overall sample level (*n* = 265), the mean global PSQI score was M = 7.79 ± 3.53 (observed range: 0.00–19.00). Overall, 72.8% of participants (*n* = 193) markedly exceeded the standard clinical cutoff threshold (>5.0), indicating a high prevalence of poor overall sleep quality across the student cohort.

Detailed analysis of the individual PSQI subscales revealed that daytime dysfunction (M = 1.74 ± 0.94; 57.0% inadequate) and sleep latency (M = 1.52 ± 1.02; 47.2% inadequate) represented the most severely impaired sleep parameters among students. Conversely, use of sleep medication (M = 0.36 ± 0.79; 89.4% adequate) and habitual sleep efficiency (M = 0.68 ± 0.98; 80.8% adequate) demonstrated the lowest levels of disruption.

**Figure 4:**
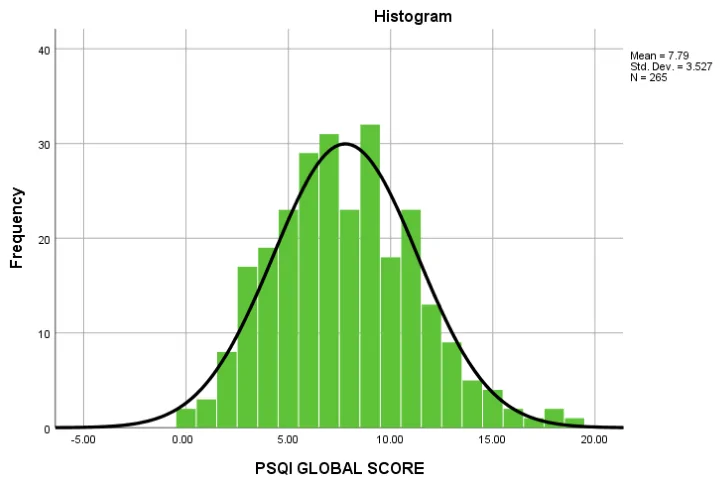
Distribution of the global PSQI score at the sample level (*n* = 265, M = 7.79 ± 3.53). The continuous black curve represents the theoretical normal distribution curve.

The overall distribution of global PSQI scores across the cohort is illustrated in Figure 4.

Bivariate comparative analysis using Pearson’s chi-square (chi^2^) tests established no statistically significant gender disparities across any of the seven individual PSQI components or the global sleep quality classification (*p* > 0.05 for all comparisons). Specifically, clinically significant poor sleep quality (>5.0) was reported at similarly elevated rates by both male (70.6%, *n* = 48) and female (73.6%, *n* = 145) students (chi^2^ = 0.232, df = 1, *p* = 0.630).

Comprehensive descriptive metrics, severity distributions, and gender-stratified comparisons across all PSQI sleep domains are systematically detailed in Table 2.

### 2.4. Correlation Analysis

To examine the correlations between the psychometric scales and demographic variables, Spearman’s rank-order correlation (r_s_) and Chi-Square (chi^2^) tests of independence were utilized. The resulting correlation matrix for the psychometric instruments is systematically detailed in Table 3.

**Figure 5:**
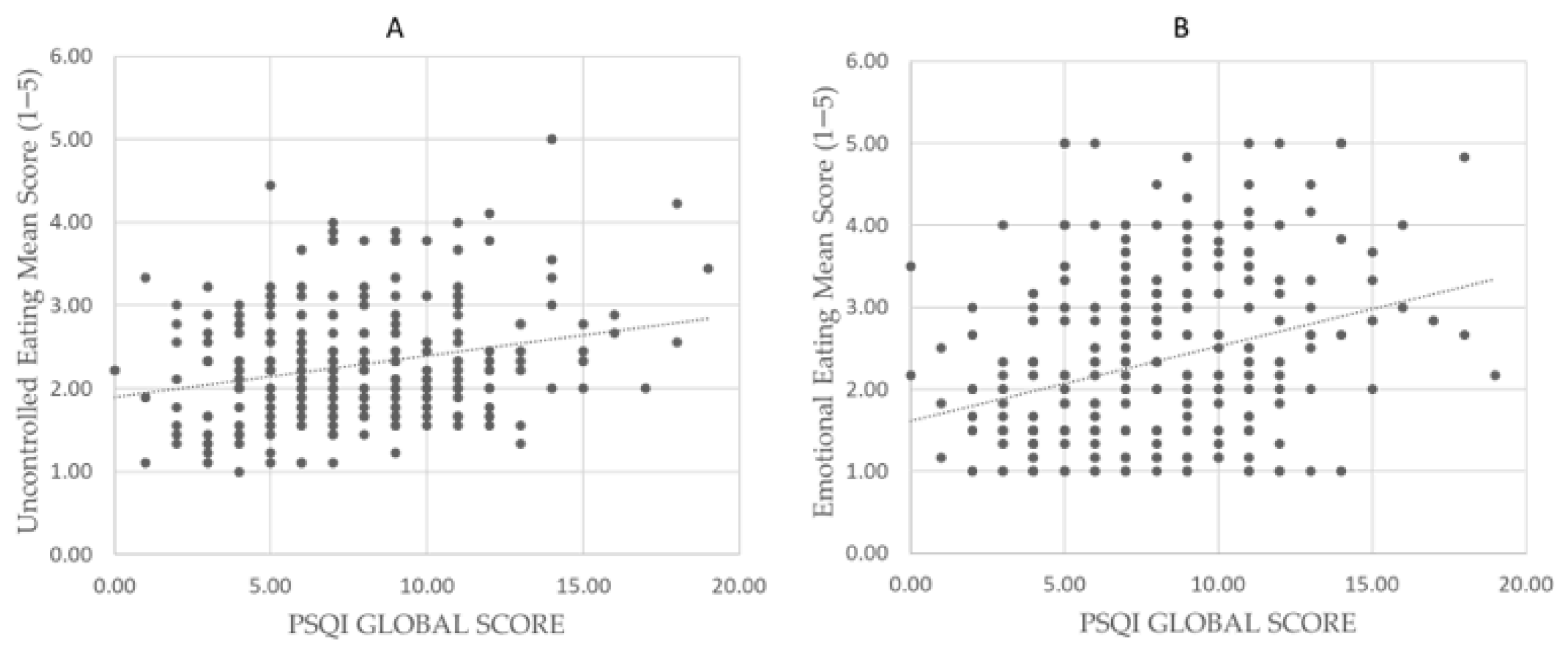
Two-dimensional scatter plots illustrating bivariate relationships between global sleep quality and eating behaviors: (**A**) Global PSQI score and Uncontrolled Eating mean score (r_s_ = 0.219, *p* < 0.001); (**B**) Global PSQI score and Emotional Eating mean score (r_s_ = 0.291, *p* < 0.001). Global PSQI scores range from 0 to 21 (with scores > 5.0 indicating clinically significant poor sleep quality). TFEQ-R21 subscales are scored on a continuous mean scale ranging from 1.00 to 5.00, where higher scores reflect greater behavioral manifestation/severity. Individual data points represent participant scores (*n* = 265 in panel A; *n* = 263 in panel B due to pairwise deletion for isolated missing items). Dotted lines depict fitted ordinary least squares (OLS) linear trendlines illustrating the positive direction of the associations.

The statistical analysis revealed significant positive correlations across all analyzed psychometric dimensions. Most notably, the PSQI Global Score co-varied positively with all three eating behavior components, demonstrating a consistent alignment between higher sleep disturbances and more problematic eating patterns. Specifically, a weak-to-moderate positive correlation was observed between sleep disturbances and Emotional Eating (r_s_ = 0.291, *p* < 0.001, Figure 5A). Furthermore, the PSQI Global Score tracked positively with Uncontrolled Eating (r_s_ = 0.219, *p* < 0.001, Figure 5B) and, weakly, with Cognitive Restraint (r_s_ = 0.141, *p* = 0.022), an association of marginal magnitude that warrants cautious interpretation given the number of bivariate tests conducted.

When evaluating the seven individual PSQI components, specific sleep impairments demonstrated differential associations across eating behavior constructs. Daytime dysfunction emerged as the strongest correlate of dietary disinhibition, associating positively with both emotional eating (r_s_ = 0.308, *p* < 0.001) and uncontrolled eating (r_s_ = 0.254, *p* < 0.001). Pharmacological sleep-aid use co-varied significantly across all measured dietary dimensions, correlating with emotional eating (r_s_ = 0.220, *p* < 0.001), cognitive restraint (r_s_ = 0.206, *p* = 0.001), and uncontrolled eating (r_s_ = 0.190, *p* = 0.002). A similar broad association was observed for prolonged sleep latency, which correlated with emotional eating (r_s_ = 0.183, *p* = 0.003), cognitive restraint (r_s_ = 0.140, *p* = 0.023), and uncontrolled eating (r_s_ = 0.138, *p* = 0.024). In addition, emotional eating was selectively linked to nocturnal sleep disturbances (r_s_ = 0.184, *p* = 0.003) and shorter perceived sleep duration (r_s_ = 0.172, *p* = 0.005), whereas poor subjective sleep quality exhibited weak associations with both uncontrolled (r_s_ = 0.145, *p* = 0.018) and emotional eating (r_s_ = 0.140, *p* = 0.022). In contrast, habitual sleep efficiency was not significantly associated with any eating behavior domain (*p* > 0.05).

Regarding the internal dimensions of the eating behavior scale, a strong and highly significant positive correlation was identified between Emotional Eating and Uncontrolled Eating (r_s_ = 0.698, *p* < 0.001). Additionally, Cognitive Restraint was associated with higher levels of both Uncontrolled Eating (r_s_ = 0.234, *p* < 0.001) and Emotional Eating (r_s_ = 0.301, *p* < 0.001). Collectively, these internal intercorrelations pointed toward a complex behavioral pattern where rigid dietary restriction frequently coexists with episodes of disinhibited eating within the studied student population.

Regarding demographic influences, age displayed significant, positive linear correlations with all three dietary behavior dimensions. Specifically, older age was associated with higher scores for Cognitive Restraint (r_s_ = 0.211, *p* = 0.001), Emotional Eating (r_s_ = 0.158, *p* = 0.010), and Uncontrolled Eating (r_s_ = 0.152, *p* = 0.013). These positive coefficients indicated that as the age of the respondents increases, there is a concurrent, statistically significant increase across all measured eating behavior scores, reflecting an amplification of both intentional dietary control mechanisms and psychological vulnerabilities toward eating disinhibition as students progress through their training. Conversely, global sleep quality was found to be independent of the participants’ age, showing no statistically significant relationship (r_s_ = −0.035, *p* = 0.572).

### 2.5. Comparative Analysis

A series of Mann–Whitney U tests highlighted significant psychometric variations tied to academic coping behaviors and lifestyle choices as systematically detailed in Table 4.

Substantial differences in eating behaviors and sleep patterns emerged when evaluating participants based on their history of weight-loss dieting and their underlying motivation for pursuing a healthy diet.

Students with a prior history of following weight-loss diets demonstrated significantly elevated levels of intentional food monitoring and psychological eating disinhibition compared to non-dieters. Specifically, students who had undertaken weight-loss diets reported markedly higher Cognitive Restraint scores, Uncontrolled Eating scores, and Emotional Eating scores compared to non-dieters. However, global sleep quality did not differ significantly between dieters and non-dieters.

Similarly, primary cognitive orientation regarding healthy eating—distinguishing between health promotion and weight reduction- yielded pronounced behavioral variations. Students who conceptualized a healthy diet primarily through the lens of weight loss exhibited significantly higher Cognitive Restraint, Uncontrolled Eating, and Emotional Eating scores. Global sleep disturbances showed a statistical trend toward higher scores among weight-loss-oriented students, though this variation did not reach formal statistical significance.

Evaluating psychometric parameters based on residential background revealed that urban students exhibited higher Cognitive Restraint scores compared to their rural counterparts. However, no statistically significant variations emerged between urban and rural residents regarding global sleep disturbances, Uncontrolled Eating, or Emotional Eating.

When evaluating the impact of housing status during the study (rented apartment, dormitory, or living with parents), a Kruskal–Wallis H test revealed a statistically significant difference across accommodation groups exclusively for Emotional Eating (H(2) = 10.400, *p* = 0.006). Post-hoc pairwise comparisons using Dunn’s test with Bonferroni adjustment demonstrated that students living in dormitories (Mdn = 2.50, IQR = 1.83; z = −2.948, *p* = 0.003, padj = 0.010) and those living in rented apartments (Mdn = 2.17, IQR = 1.50; z = −2.846, *p* = 0.004, padj = 0.013) reported significantly higher Emotional Eating scores compared to students residing with their parents (Mdn = 1.83, IQR = 1.17).

In contrast, scores did not differ significantly between students in dormitories and those in rented apartments (z = 0.680, *p* = 0.497, padj = 1.000). No statistically significant differences were observed across housing categories regarding global sleep quality (H(2) = 0.223, *p* = 0.894), Cognitive Restraint (H(2) = 4.192, *p* = 0.123), or Uncontrolled Eating (H(2) = 5.512, *p* = 0.064).

To examine sociodemographic variations in nocturnal eating habits and weight-loss dieting history among healthcare university students, cross-tabulated Chi-Square (chi^2^) tests of independence were conducted (*n* = 265).

Regarding late-night food intake (eating after 21:00), a statistically significant association emerged with gender (chi^2^(1) = 7.564, *p* = 0.006). Male students demonstrated a substantially higher prevalence of late-night food consumption compared to female students (77.9% vs. 59.4%). In contrast, late-night eating showed no statistically significant associations with residential background (chi^2^1) = 0.044, *p* = 0.834; 64.6% urban vs. 63.4% rural), current housing status during studies (chi^2^(2) = 1.203, *p* = 0.548), or academic year of study (chi^2^(8) = 8.644, *p* = 0.373).

When evaluating the history of weight-loss dieting, residential background was identified as the sole significant sociodemographic factor (chi^2^(1) = 5.213, *p* = 0.022). Specifically, students originating from urban areas were significantly more likely to report a prior history of weight-loss diets compared to their rural counterparts (42.7% vs. 28.7%). Conversely, no statistically significant associations were found between a history of weight-loss dieting and gender (chi^2^(1) = 0.979, *p* = 0.322; 39.1% females vs. 32.4% males), current housing status during the study (chi^2^(2) = 0.602, *p* = 0.740), or academic year (chi^2^(8) = 15.102, *p* = 0.057).

### 2.6. Regression Analysis

To evaluate the combined predictive value of eating behavior dimensions, global sleep quality, sex, and age on the likelihood of late-night food intake (eating after 21:00), a binary logistic regression model was constructed (*n* = 265, see Table 5).

The overall multivariate model was highly statistically significant, chi^2^(6) = 45.489, *p* < 0.001, explaining 21.6% of the variance in late-night eating behavior (Nagelkerke R^2^ = 0.216; Cox & Snell R^2^ = 0.158), with an overall correct classification rate of 74.3% (Hosmer and Lemeshow chi^2^(8) = 8.988, *p* = 0.343).

Conversely, dietary disinhibition significantly elevated the likelihood of nocturnal eating. Higher Uncontrolled Eating scores nearly doubled the odds of consuming food late at night (B = 0.656, SE = 0.306, Wald = 4.613, *p* = 0.032, OR = 1.928, 95% CI [1.059, 3.508]).

Furthermore, poorer sleep hygiene was confirmed as an independent contributor; each one-point increase in the Global PSQI Score was linked to a 12.1% increase in the odds of late-night eating (B = 0.114, SE = 0.045, Wald = 6.393, *p* = 0.011, OR = 1.121, 95% CI [1.026, 1.225]).

Demographic variables also demonstrated significant independent associations within the model. Female students were 61.5% less likely to report late-night food intake compared to male respondents (B = −0.956, SE = 0.352, Wald = 7.366, *p* = 0.007, OR = 0.385, 95% CI [0.193, 0.767]).

Additionally, age exhibited a marginal negative association, with each additional year of age associated with a 6.2% reduction in the odds of eating after 21:00 (B = −0.063, SE = 0.032, Wald = 3.853, *p* = 0.050, OR = 0.938, 95% CI [0.881, 1.000]).

When controlling for uncontrolled eating, cognitive restraint, sleep quality, sex, and age, the Emotional Eating subscale did not reach statistical significance in predicting late-night food consumption (*p* = 0.874). This lack of statistical significance reflects the substantial collinearity and shared variance between emotional eating and uncontrolled eating (r_s_ = 0.698), with the latter capturing the behavioral disinhibition driving late-night food intake in the multivariable model, rather than indicating an absence of emotional mechanisms.

### 2.7. Mediation Analysis

**Figure 6:**
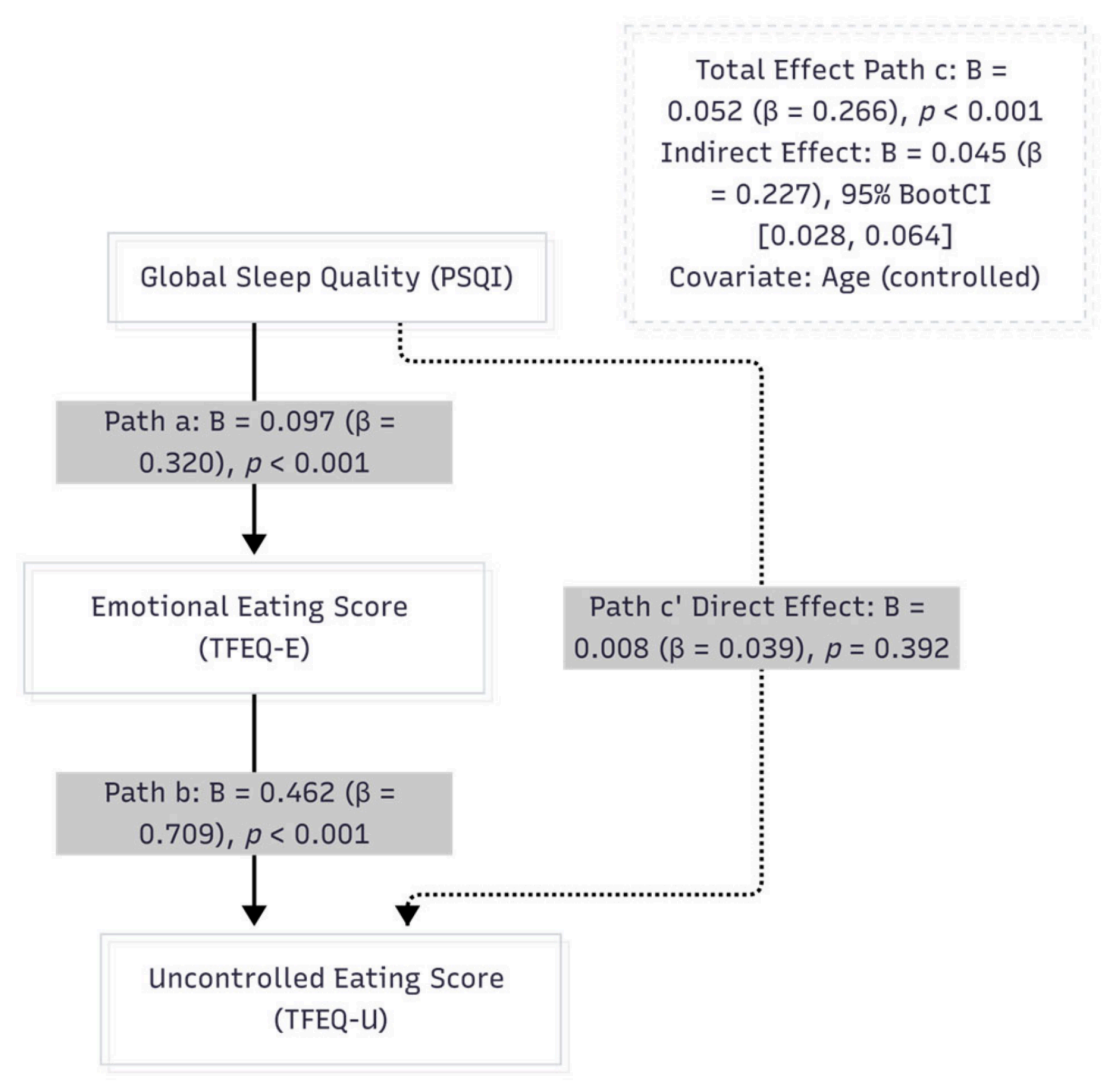
Mediation model evaluating the relationship between global sleep quality and uncontrolled eating, with emotional eating acting as the mediator. B = unstandardized regression coefficient; *p* = statistical significance level. Solid lines indicate statistically significant pathways (*p* < 0.001), while the dotted line indicates a statistically non-significant direct pathway (*p* = 0.392), indicating a significant indirect association between sleep quality and uncontrolled eating via emotional eating, after controlling for age.

To test whether emotional eating accounts for the relationship between poor sleep quality and uncontrolled eating, a mediation analysis was conducted using Model 4 of Hayes’ PROCESS macro with 5000 bootstrap resamples, controlling for participant age (*n* = 265, see Figure 6).

In the first step, global sleep quality significantly and positively predicted emotional eating (Path a: B = 0.0967, SE = 0.0178, *t* = 5.427, *p* < 0.001, 95% CI [0.0616, 0.1318], β = 0.3195), with the model accounting for 10.87% of the variance in emotional eating (R^2^ = 0.1087, F(2, 262) = 15.973, *p* < 0.001).

The covariate age also showed a significant positive association with emotional eating (B = 0.0325, SE = 0.0141, *t* = 2.304, *p* = 0.022, β = 0.1356).

In the second step, when controlling for sleep quality and age, emotional eating emerged as a strong positive predictor of uncontrolled eating (Path b: B = 0.4621, SE = 0.0296, *t* = 15.635, *p* < 0.001, 95% CI [0.4039, 0.5203], β = 0.7090). Collinearity diagnostics confirmed acceptable discriminant validity between emotional eating and uncontrolled eating (VIF = 1.122, Tolerance = 0.891).

Age was not a significant predictor of uncontrolled eating in the multivariate model (B = 0.0009, SE = 0.0068, *t* = 0.138, *p* = 0.891, β = 0.0060).

The total effect of sleep quality on uncontrolled eating was statistically significant (Path c: B = 0.0524, SE = 0.0118, *t* = 4.425, *p* < 0.001, 95% CI [0.0291, 0.0757], β = 0.2656). After introducing emotional eating into the model, the direct association between sleep quality and uncontrolled eating was not statistically significant (Path c’: B = 0.0077, SE = 0.0090, *t* = 0.857, *p* = 0.392, 95% CI [−0.0100, 0.0254], β = 0.0390). The overall regression model accounted for 52.16% of the variance in uncontrolled eating (R^2^ = 0.5216, F(3, 261) = 94.861, *p* < 0.001); however, this variance was driven almost entirely by path b (emotional eating), whereas the direct contribution of sleep disturbances on path a was considerably smaller (R^2^ = 0.1087). The completely standardized indirect effect corresponded to β = 0.2265 (95% BootCI [0.1476, 0.3090]).

The indirect effect was statistically significant, as the 95% percentile bootstrap confidence interval did not include zero (unstandardized indirect effect: Effect = 0.0447, BootSE = 0.0090, 95% BootCI [0.0282, 0.0636]; completely standardized indirect effect: β = 0.2265, BootSE = 0.0413, 95% BootCI [0.1476, 0.3090]). These results indicate that the association between sleep disturbances and uncontrolled eating is largely accounted for by indirect covarying mechanisms through emotional eating, while the direct pathway remained statistically unconfirmed.

## 3. Discussion

The research results showed that healthcare students had a poor sleep quality level. Global sleep quality was not significantly associated with participants’ age (r_s_ = −0.035, *p* = 0.572), suggesting that poor sleep quality represents a pervasive and chronic concern across healthcare education, regardless of students’ age or academic seniority.

More than half of participants manifest a functional and stable relationship with food, identifying low scores for cognitive restriction (52.8%), uncontrolled eating (63.8%) and emotional eating (58.1%). This shows that over half of the sample manifests optimal regulation for all 3 subscales considered. The area with moderate manifestation is cognitive restriction (38.1%). This result can be interpreted as reflecting the awareness of a proper diet among medical students, based on the knowledge accumulated over the years of study. Another explanation could be the interest in body image and the desire to adopt preventive behaviors. However, a subset of students exhibited elevated behavioral vulnerability. The analysis of the subscales shows that emotional eating registers the highest prevalence in the upper manifestation tier (11.7%, *n* = 31), exceeding the proportion observed for high food disinhibition (5.3%). This result can be explained by academic stress and anxiety related to school activities, as well as the challenges of student life (time and money management, housing, schedule, exam periods, competition, contact with patients).

The study identified a significant positive correlation between the Global PSQI score and all eating behavior subscale scores. This finding suggests that sleep disturbances may destabilize students’ overall psychological relationship with food by weakening dietary self-regulation, increasing emotionally driven cravings, and contributing to loss-of-control overeating.

The analysis of data highlighted that late-night eating is independently predicted by a combination of lower cognitive restraint, high uncontrolled eating, poor sleep quality, male gender and younger age. More explicitly, predictors for late-night snacks are: Cognitive restraint as a protective factor, Uncontrolled eating as a risk factor, poor sleep quality as a physiological driver, male gender, and younger age as socio-demographic factors.

The findings provide a clear psychological and physiological profile of late-night eating among students. Nocturnal snacking does not appear to result merely from skipped meals; rather, it is strongly associated with impaired impulse regulation, reflected in uncontrolled eating, and with disrupted biological rhythms, reflected in poor sleep quality. In contrast, intentional cognitive control over food choices may function as an important protective factor against late-night cravings. Moreover, male students appear to be significantly more susceptible to late-night eating than female students.

When analysing psychometric profiles across genders, no significant differences were identified for the global sleep score; this result supports the fact that male and female healthcare students are equally affected by sleep problems. The results in the literature regarding medical students are similar; however, some research has highlighted the fact that, among the general population, women have reported sleeping shorter times or slightly less efficiently than men [1].

In our study, female students reported significantly higher scores for Emotional Eating compared to male counterparts but at the same time exhibited a lower risk for late-night food consumption. These results are consistent with the literature, which suggests that women are more prone to emotional eating, meaning eating as a response to negative emotions or stress [22]. During the day, women are more prone to emotion-driven eating due to stress, overload or dealing with daily tasks. Several studies identified the same results in different university student populations: in Japan, Emotional eating was found to be related to the ideal body shape in normal-weight females [23], while in Russia, Budkevich et al. [24] showed that cognitive eating restraint and uncontrolled eating among female university students were related to the morning subscale, while emotional eating was related to the evening subscale. In Poland, Grajek et al. [10] identified that students with high scores for emotional eating were more likely to underestimate the size and caloricity of the meal, while Alexatou et al. [25] showed that female university students with emotional eating were more prone to experience psychological distress.

However, our results showed that this emotional vulnerability does not translate into nocturnal dietary disinhibition. The fact that female students avoid late-night meals can be explained by several factors: women are more likely to be concerned about body image and weight in order to conform to a socio-cultural model, which makes them more careful about their diet. Comparative literature highlighted that male students are more prone to adopt risky behaviors than female students when it comes to impulsive and risky behaviors. For example, Ferrara et al. [26] identified that sleep deprivation causes a decrease in risky choices in females and an increase in selfishness. Qian et al. [27] identified that males had elevated cravings for energy-dense and savory foods when they were sleep-deprived. Akhlaghi & Kohanmoo [28] showed that poor sleep is associated with emotional eating and high BMI among female students. Du et al. [29] also focused on gender differences and identified that uncontrolled and emotional eating mediated the relationship between perceived stress and dietary risk, and higher sleep quality weakened this relationship among female university students but not males. These gender differences, determined by biological, neurological and psychological aspects related to eating and sleeping behavior, are extremely important when analyzing the pathways through which such behaviors are achieved but also when structuring interventions regarding evening eating or weight gain.

When analysing psychometric profiles across environmental backgrounds, we identified that healthcare students living in urban areas are more likely to exhibit Cognitive restraint than students from rural areas. Comparative analysis also showed that urban students are more prone to have a history of weight-loss diets. The results could also be related to the body-image cultural model among young people in larger cities and the pressure for satisfaction with body image. In big cities, there are more body care offers (fitness halls, sports facilities, medical and aesthetic services, etc.) that students can benefit from, as well as high social pressure for a healthy body. Similar results were identified by Zameer et al. [30], who showed that urban students are more prone to stress due to diverse activities, busy schedules, distances, and other urban-related factors.

Comparative analysis showed that students living in dormitories or rented apartments use food to cope with their emotions significantly more often than students living with their families. This finding may be explained by several factors. First, students who leave their family homes may be more vulnerable to psychological distress associated with living independently. Second, they may have less access to regular meals and home-cooked food, which can contribute to meal skipping and greater reliance on fast food. Finally, living in dormitories or shared apartments may increase exposure to late meals, snacking, and recreational eating with roommates or peers. The findings are similar to those obtained by Lawson et al. [31] in medical university students in Ghana and Yildirim et al. [32] in Türkiye. The researchers found that students who stay home alone and students who stay with their family have higher sleep quality, while those who stay home with friends have lower sleep quality.

The mediation model indicates that poor sleep quality does not lead directly to uncontrolled eating among medical students through purely physiological appetite mechanisms. Instead, poor sleep appears to weaken emotional regulation, increasing vulnerability to emotion-driven eating, which then contributes to uncontrolled food intake. These findings suggest that uncontrolled eating is shaped by the interaction of physiological disruption and emotional vulnerability. In this context, poor sleep may not directly increase hunger, but it may make students more susceptible to stress and reduce their capacity to resist maladaptive eating responses. These results suggest that stress-related physiological and neurological vulnerability may increase students’ susceptibility to negative affect. This finding is relevant for students, academic counseling staff, and clinicians alike, as it highlights the need to address sleep hygiene, stress-coping strategies, psychological empowerment, and the promotion of a healthy lifestyle, rather than focusing exclusively on weight control or dietary restriction.

Our findings align with a growing body of literature highlighting the central role of emotional eating as an indirect pathway across diverse psychological antecedents and dysregulated eating outcomes. Specifically, recent investigations have similarly identified emotional eating as a key mediating mechanism linking higher-order psychological vulnerabilities to uncontrolled and compulsive eating behaviors [33,34]. While the ubiquity of emotional eating as a mediator raises valid considerations regarding whether it captures a distinct psychological process or shared construct variance with the outcome, our collinearity diagnostics and the moderate-to-strong subscale inter-correlation remained below the conventional threshold for psychometric redundancy, supporting the discriminant validity of emotional and uncontrolled eating within our cohort.

### 3.1. Strengths and Limitations of the Study

A key strength of this research is its identification of a significant indirect mediating pathway linking sleep disorders to uncontrolled food intake through emotional eating, controlling for age. In addition, the study outlines the psychometric profile of medical students, a population particularly vulnerable to poor sleep quality and eating-related difficulties due to the demands of academic training, including prolonged stress, intensive schedules, irregular meal patterns, night shifts, and frequent exposure to patients’ suffering.

Although the current study provides valuable insights into the mechanisms linking sleep quality to uncontrolled eating, several limitations should be acknowledged. First, the cross-sectional design prevents the establishment of temporal precedence or causal relationships, particularly regarding the proposed mediation pathway from global sleep quality through emotional eating to uncontrolled eating. Because data were collected concurrently, the direction of effects cannot be definitively adjudicated, and a statistically reversed specification (with uncontrolled eating preceding emotional eating) yields comparable explanatory power. Longitudinal studies would be needed to examine these variables over time and clarify the directionality of these associations.

Second, another limitation is due to the absence of anthropometric indices, standardized mental health metrics, and detailed dietary histories. Objective height, weight, and body mass index (BMI) were not recorded, although eating behavior scores covary with body mass and a history of dieting is inherently intertwined with weight status, leaving potential for residual confounding. Furthermore, while dieting history was captured categorically, long-term dietary patterns and prior nutritional trajectories were not evaluated. Similarly, although the theoretical framework highlights academic stress, anxiety, and burnout, psychological distress was not directly quantified using validated mental health scales. Consequently, these unmeasured variables could not be controlled for within the mediation model, leaving open the possibility that emotional eating may partly operate as a proxy for broader psychological distress or established dietary habits, which represents a plausible common antecedent to both impaired sleep and maladaptive eating behaviors.

Third, the study relied on self-report instruments, which are subject to recall bias and social desirability. Relatedly, emotional eating and uncontrolled eating were assessed via subscales of the same instrument (r_s_ = 0.698), meaning that shared method and construct variance may have contributed to the strength of their association.

Fourth, the TFEQ-R21 was administered using an adapted 5-point Likert scale with mean-score scoring rather than the original 4-point structure and 0–100 transformation; consequently, the tertile score bands (Low, Moderate, High) represent descriptive sample heuristics rather than clinically validated risk cut-offs.

Fifth, the generalizability of the findings is limited by the non-probability snowball sampling method, which precluded calculating a formal response rate, and an a priori power analysis was not performed, although the achieved sample size (*n* = 265) provided adequate empirical power for the multivariable models evaluated. Future research should therefore include larger, randomly selected cohorts and samples from the general population, different age groups, and diverse cultural contexts in order to better understand how body-image ideals and eating-related behaviors may vary across populations.

Additionally, late-night food intake was captured via a single binary item (cutoff at 21:00) without assessing meal frequency, nutritional composition, individual chronotype, or clinical shift work patterns.

### 3.2. Practical Implications

During their university training, medical students gradually acquire more knowledge about nutrition, dietary behavior, and the effects of eating patterns on physical and mental health. They also become more familiar with diets and their associations with chronic disease prevention and management. As a result, students may place greater emphasis on controlling food intake than on regulating the emotional factors that influence eating behavior. This distinction is important for counseling interventions, which should address not only nutritional knowledge and dietary control but also emotional self-regulation.

These findings can inform counseling interventions for health sciences students. Strategies focused solely on weight-loss dieting or strict calorie control may be ineffective and could worsen dysfunctional eating behaviors. Instead, interventions should emphasize psychological support, improved sleep quality, and emotion-regulation skills, helping students shift from rigid weight-control practices toward a healthier, more balanced lifestyle.

## 4. Materials and Methods

### 4.1. Participants and Study Design

A cross-sectional study was conducted between December 2025 and May 2026, among students registered at “Grigore T. Popa” University of Medicine and Pharmacy in Iași and the Faculty of Medicine and Pharmacy, University of Oradea, Romania.

A non-probability snowball sampling strategy was utilized to recruit participants across multiple health science fields, explicitly including General Medicine, Dentistry, Pharmacy, Nursing, Physiotherapy, and Nutrition and Dietetics, encompassing all years of undergraduate study, ages, and genders.

The online questionnaires were initially shared via digital platforms with student representatives responsible for coordinating specific academic years. These representatives acted as primary seeds to initiate the recruitment chain, subsequently disseminating the survey link among their peers and encouraging them to further forward the questionnaire to other eligible students within their respective networks. Due to the chain-referral nature of digital snowball sampling, the total number of individuals reached could not be monitored, precluding the calculation of a definitive response rate. Completing the questionnaire was completely voluntary and anonymous, and proceeding past the introductory interface implied informed consent to participate in the study. Prior to initiating the survey, students were thoroughly informed about the explicit purpose of the investigation, the guaranteed confidentiality of their raw responses, and their absolute right to withdraw from the study at any given time without incurring any negative consequences. No material or academic incentives were offered to the respondents.

The inclusion criteria required participants to hold an active student status within the target faculties during the data collection window. The exclusion criteria were incomplete fill-in forms and questionnaires submitted after the deadline. Students self-reporting health conditions, active medical treatments, medication use, or specific dietary regimens were not formally screened out or excluded, as the study aimed to assess a naturalistic cross-section of healthcare students; however, sleep medication usage was captured within the PSQI protocol and history of weight-loss dieting was explicitly evaluated as an analytical variable. The final analytical sample comprised 265 participants, with pairwise deletion applied for isolated missing item responses on individual subscales (yielding *n* = 263 for Emotional Eating).

### 4.2. Research Tool

Data were collected through an online questionnaire, developed using the Google Forms platform. The instrument was structured in four main sections, as follows:

- The first section recorded socio-demographic and academic variables, such as gender, age, background environment (urban vs. rural), residential status during studies, and the current academic year. Further items evaluated late-night eating habits (after 9 PM) and history of dieting periods.
- The second section measured eating behaviors and psychological profiles using the Three-Factor Eating Questionnaire (TFEQ-R21) [35]. This included 21 items targeting three independent dimensions: Cognitive Restraint (6 items evaluating intentional food monitoring), Uncontrolled Eating (9 items measuring the loss of dietary control), and Emotional Eating (6 items assessing affect-triggered food intake). The items were rated on a 5-point Likert scale, ranging from 1 (“Never”) to 5 (“Always”). In accordance with adapted scoring protocols for uniform 5-point matrices, subscale metrics were calculated using the Mean Score method (calculating the arithmetic mean of items within each respective subscale), yielding continuous scores ranging from 1.00 to 5.00. Higher mean scores indicate a more pronounced manifestation or greater severity of the respective eating behavior dimension. Subscale scores were subsequently categorized into three heuristic manifestation tiers based on equal tertile division of the 1.00–5.00 continuum: Low (1.00–2.33), Moderate (2.34–3.66), and High (3.67–5.00). These bands were utilized strictly as a descriptive heuristic to characterize sample distribution rather than as clinically anchored diagnostic thresholds. Psychometric evaluation demonstrated outstanding internal consistency across all three constructs, yielding Cronbach’s alpha coefficients of α = 0.910 for Cognitive Restraint, α = 0.879 for Uncontrolled Eating, and α = 0.948 for Emotional Eating, indicating excellent internal consistency and structural reliability for the assessment tool.
- The third section measured global sleep quality and underlying sleep architecture using the Pittsburgh Sleep Quality Index (PSQI) [36]. This included 19 individual items that generated seven core component scores: subjective sleep quality, sleep latency, sleep duration, habitual sleep efficiency, sleep disturbances, use of sleep medication, and daytime dysfunction. Each component is rated on a 4-point scale ranging from 0 to 3. The global PSQI score is calculated by aggregating these seven components, resulting in a total score ranging from 0 to 21 points. In terms of clinical interpretation, higher global scores reflect poorer sleep quality, with a total score of 5 or less (≤5) indicating good sleep quality, and a score greater than 5 (>5) serving as the internationally validated diagnostic cutoff threshold to identify “poor sleepers” exhibiting significant sleep disturbances. Following the analysis, Cronbach’s alpha was 0.683 for the core components, indicating acceptable internal consistency for the assessment tool.

### 4.3. Statistical Analysis

Data processing and analysis were performed using IBM Statistical Package for the Social Sciences (SPSS) for Windows, version 26 (SPSS Inc., Chicago, IL, USA) and Hayes’ PROCESS macro (v5.0). Continuous variables were described by means (M) and standard deviations (SD), or medians (Mdn) and interquartile ranges (IQR) where appropriate, and categorical variables by absolute and relative frequencies (percentages). Sample sizes fluctuated across specific analyses due to incomplete or optional responses. A pairwise deletion approach was implemented to handle missing observations and preserve maximum statistical power. Given the exploratory nature of the secondary bivariate tests, unadjusted findings near the significance threshold were interpreted with caution. Effect sizes for non-parametric two-group comparisons were calculated as r = |z|/√N.

Data normality was assessed using the Kolmogorov–Smirnov test, which revealed that continuous and ordinal variables, specifically age, average sleep hours, global sleep quality (PSQI) scores, and the three standardized subscales of the Three-Factor Eating Questionnaire (TFEQ-R21), did not follow a normal distribution (*p* < 0.05). Consequently, non-parametric statistics were applied, and homogeneity of variances was evaluated using Levene’s test prior to conducting comparative analyses. Bivariate associations between non-normally distributed continuous or ordinal metrics were evaluated using Spearman’s rank correlation coefficient (r_s_), while Chi-Square (chi^2^) (with Yates’ continuity correction where applicable) tests of independence were utilized to examine cross-tabulated associations between categorical demographic background parameters (gender, residential background) and behavioral outcomes (late-night food intake, history of weight-loss diets). The Mann–Whitney U test was used to compare two independent groups (e.g., urban vs. rural). For comparisons involving more than two independent groups (housing status: rented apartment vs. dormitory vs. living with parents), the Kruskal–Wallis H test was conducted, followed by pairwise post-hoc evaluations.

The internal consistency and psychometric reliability of the global PSQI components and the TFEQ-R21 subscales were evaluated using Cronbach’s alpha coefficient (α).

Predictors for the binary logistic regression model were selected based on theoretical relevance and preliminary significant bivariate associations (*p* < 0.05). Categorical variables were dummy-coded (0 = absence/reference group, 1 = presence). Multicollinearity was assessed using Variance Inflation Factors (VIF), with all values remaining below 2.0, indicating the absence of significant collinearity. Model goodness-of-fit and performance were evaluated using the Omnibus test of model coefficients (*p* < 0.05), the Hosmer and Lemeshow goodness-of-fit test, as well as Cox & Snell and Nagelkerke pseudo-R^2^ statistics. Specifically, a binary logistic regression model was executed to evaluate late-night eating habits (after 21:00) based on eating behavior subscales (Cognitive Restraint, Uncontrolled Eating, Emotional Eating), global sleep quality (PSQI), sex, and age. For the logistic regression models, unstandardized regression coefficients (B), standard errors (S.E.), Wald statistics, significance levels (*p*), and Odds Ratios (OR) with their corresponding 95% Confidence Intervals (CI) were calculated.

Furthermore, to evaluate the structural pathways linking sleep disturbances to altered eating behaviors, a mediation analysis was conducted using Model 4 of Hayes’ PROCESS macro (v5.0) with 5000 bootstrap resamples to compute 95% percentile bootstrap confidence intervals. Both unstandardized (B) and completely standardized (β) coefficients are reported. Participant age was entered as a statistical covariate to control for confounding identified in bivariate analyses. Discriminant validity between the TFEQ-R21 subscales was confirmed via collinearity diagnostics (VIF = 1.122, Tolerance = 0.891).

### 4.4. Ethical Approval

The research adhered to the ethical principles set out in the Declaration of Helsinki. The study was approved by the Research Ethics Committee of the University of Medicine and Pharmacy, Oradea, Romania, No. 21129/22.12.2025.

## 5. Conclusions

These findings suggest that poor sleep quality may activate an emotional eating pathway, which in turn contributes to reduced dietary control. Rigid cognitive restraint and weight-loss-oriented control may further increase psychological vulnerability. Therefore, campus health interventions should move beyond restrictive dietary approaches and instead promote psychological balance, stress management, sleep hygiene, and emotion-regulation skills. Such strategies may help healthcare students better manage academic challenges and adopt healthier lifestyles during their university years.

## Figures and Tables

**Table 1:** Psychometric properties and descriptive statistics of the TFEQ-R21 standardized subscales ^1^.

TFEQ-R21 Subscale	Cronbach’s Alpha (α)	Mean (M)	Std. Dev. (SD)
Cognitive Restraint	0.910	2.38	0.956
Uncontrolled Eating	0.879	2.28	0.695
Emotional Eating	0.948	2.33	1.067

^1^ Sample size was *n* = 265 for Cognitive Restraint and Uncontrolled Eating, and *n* = 263 for Emotional Eating due to isolated missing items.

**Table 2:** Descriptive statistics and gender comparison for PSQI global score and component subscales (*n* = 265) ^1^.

PSQI Components	Total Sample M ± SD	Total (*n* = 265)	Male (*n* = 68)	Female (*n* = 197)	chi^2^	*p*-Value
Subjective sleep quality	1.25 ± 0.74				0.009	0.923
Adequate		67.2%	67.6%	67.0%		
Inadequate		32.8%	32.4%	33.0%		
Sleep latency	1.52 ± 1.02				0.092	0.762
Adequate		52.8%	54.4%	52.3%		
Inadequate		47.2%	45.6%	47.7%		
Sleep duration	0.94 ± 0.81				0.821	0.365
Adequate		78.9%	75.0%	80.2%		
Inadequate		21.1%	25.0%	19.8%		
Habitual sleep efficiency	0.68 ± 0.98				1.949	0.163
Adequate		80.8%	75.0%	82.7%		
Inadequate		19.2%	25.0%	17.3%		
Sleep disturbances	1.29 ± 0.68				1.306	0.253
Adequate		66.4%	72.1%	64.5%		
Inadequate		33.6%	27.9%	35.5%		
Use of sleep medication	0.36 ± 0.79				0.999	0.317
Adequate		89.4%	92.6%	88.3%		
Inadequate		10.6%	7.4%	11.7%		
Daytime dysfunction	1.74 ± 0.94				2.666	0.103
Adequate		43.0%	51.5%	40.1%		
Inadequate		57.0%	48.5%	59.9%		
Global PSQI Score	7.79 ± 3.53				0.232	0.630
Good overall sleep quality (≤5)		27.2%	29.4%	26.4%		
Poor overall sleep quality (>5)		72.8%	70.6%	73.6%		

^1^ M ± SD = Mean ± Standard Deviation; chi^2^ = Pearson’s Chi-Square statistic (df = 1); *p*-value determined using two-tailed asymptotic significance (significance threshold at *p* < 0.05). Component scores range from 0 to 3, where scores of 0–1 indicate adequate component function and scores of 2–3 indicate inadequate function. Global PSQI score ranges from 0 to 21, where scores > 5.0 denote clinically significant poor overall sleep quality.

**Table 3:** Spearman’s rank correlation matrix between TFEQ-R21 subscale scores and PSQI global score and component subscales (*n* = 265).

Instruments	1	2	3	4	5	6	7	8	9	10
1. Cognitive Restraint Score	—									
2. Uncontrolled Eating Score	0.234 **	—								
3. Emotional Eating Score	0.301 **	0.698 **	—							
4. PSQI Global Score	0.141 *	0.219 **	0.291 **	—						
5. Subjective sleep quality	0.005	0.145 *	0.140 *	0.708 **	—					
6. Sleep latency	0.140 *	0.138 *	0.183 **	0.731 **	0.508 **	—				
7. Sleep duration	0.083	0.067	0.172 **	0.549 **	0.484 **	0.250 **	—			
8. Habitual sleep efficiency	−0.035	−0.017	−0.028	0.378 **	0.090	0.132 *	0.126 *	—		
9. Sleep disturbances	0.0101	0.116	0.184 **	0.620 **	0.421 **	0.484 **	0.175 **	0.138 *	—	
10. Use of sleep medication	0.206 **	0.190 **	0.220 **	0.431 **	0.159 **	0.289 **	0.056	−0.008	0.272 **	—
11. Daytime dysfunction	0.082	0.254 **	0.308 **	0.646 **	0.432 **	0.324 **	0.328 **	0.016	0.304 **	0.170 **

**. Correlation is significant at the 0.01 level (2-tailed), *. Correlation is significant at the 0.05 level (2-tailed). All values represent Spearman’s rank-order correlation coefficients (r_s_). Total sample size was *n* = 265, except for bivariate correlations involving Emotional Eating (*n* = 263), where pairwise deletion was applied due to isolated missing items.

**Table 4:** Non-parametric group comparisons (Mann–Whitney U tests) for eating behavior sub-scales, sleep quality, and age across behavioral and demographic categories (*n* = 265) ^1^.

Variable	Group 1 Mdn (IQR)	Group 2 Mdn (IQR)	Mann–Whitney U	z	*p*-Value	Effect Size (r)
History of weight-loss diets	Yes (*n* = 99)	No (*n* = 166)				
Cognitive Restraint	3.00 (1.17)	1.83 (1.17)	2774.00	−9.036	<0.001	0.55
Uncontrolled Eating	2.33 (1.00)	2.00 (0.89)	6000.00	−3.680	<0.001	0.23
Emotional Eating (*n* = 263)	2.67 (1.33)	1.83 (1.67)	5277.00	−4.884	<0.001	0.30
Global Sleep Quality (PSQI)	8.00 (4.00)	7.00 (5.00)	7399.50	−1.360	0.174	0.08
Healthy diet motivation	Weight loss	Health promotion				
Cognitive Restraint	2.83 (1.17)	2.00 (1.50)	4327.50	−5.148	<0.001	0.32
Uncontrolled Eating	2.33 (1.22)	2.11 (0.78)	5662.50	−2.787	0.005	0.17
Emotional Eating (*n* = 263)	2.33 (2.00)	2.00 (1.67)	6069.00	−2.069	0.039	0.13
Global Sleep Quality (PSQI)	9.00 (5.00)	7.00 (5.00)	6222.50	−1.800	0.072	0.11
Residential background	Urban (*n* = 164)	Rural (*n* = 101)				
Cognitive Restraint	2.50 (1.50)	2.00 (1.33)	5876.00	−3.979	<0.001	0.24
Uncontrolled Eating	2.22 (0.89)	2.22 (0.94)	8142.50	−0.231	0.818	0.01
Emotional Eating (*n* = 263)	2.17 (1.63)	2.17 (1.50)	8115.00	−0.276	0.782	0.02
Global Sleep Quality (PSQI)	8.00 (5.00)	7.00 (4.00)	8191.50	−0.150	0.881	0.01
Gender	F (*n* = 197)	M (*n* = 68)				
Cognitive Restraint	2.50 (1.33)	1.92 (1.50)	5710.00	−1.820	0.069	0.11
Uncontrolled Eating	2.22 (1.00)	2.17 (0.67)	6365.00	−0.610	0.540	0.04
Emotional Eating (*n* = 263)	2.17 (1.67)	1.83 (1.46)	5287.50	−2.600	0.009	0.16
Global Sleep Quality (PSQI)	8.00 (5.00)	7.00 (5.50)	6065.50	−1.170	0.244	0.07
Age across behavioral habits	Present	Absent				
Late-night food intake	21.00 (3.00)	21.00 (5.00)	6645.00	−2.429	0.015	0.15

^1^ Mdn = Median; IQR = Interquartile Range; U = Mann–Whitney U test statistic; z = standardized test statistic; *p*-value determined using two-tailed asymptotic significance; r = |z|/√*n* (effect size estimate). Total sample size was *n* = 265 across all analyses, except for Emotional Eating (*n* = 263), where two observations were missing.

**Table 5:** Logistic regression model parameters for predicting late-night eating (after 21:00) among health sciences students ^1^.

Predictor Variables	B	S.E.	Wald	df	*p*	OR (Exp B)	95% CI for OR
Cognitive Restraint Mean Score	−0.704	0.167	17.787	1	<0.001	0.494	0.356–0.686
Uncontrolled Eating Mean Score	0.656	0.306	4.613	1	0.032	1.928	1.059–3.508
Emotional Eating Mean Score	−0.032	0.202	0.025	1	0.874	0.968	0.652–1.437
Global Sleep Quality (PSQI)	0.114	0.045	6.393	1	0.011	1.121	1.026–1.225
Sex (Female)	−0.956	0.352	7.366	1	0.007	0.385	0.193–0.767
Age	−0.063	0.032	3.853	1	0.050	0.938	0.881–1.000

^1^ B = unstandardized regression coefficient; S.E. = standard error; Wald = Wald test statistic; *p* = statistical significance level; OR = Odds Ratio; CI = Confidence Interval.

## Data Availability

Data supporting the findings of this study are available from the main author upon reasonable request.

## Abbreviations

The following abbreviations are used in this manuscript:

B	Unstandardized regression coefficient
BootSE	Bootstrap Standard Error
BootLLCI	Bootstrap Lower-Level Confidence Interval
BootULCI	Bootstrap Upper-Level Confidence Interval
CI	Confidence Interval
df	Degrees of freedom
Exp(B)	Exponentiated coefficient (Odds Ratio)
M	Mean
Mdn	Median
Min	Minimum
Max	Maximum
*n*	Number of participants/Sample size
OR	Odds Ratio
*p*	Statistical significance level
r_s_	Spearman’s rank correlation coefficient
R^2^	Coefficient of determination (Nagelkerke/Cox & Snell pseudo-R-squared)
SD	Standard Deviation
SE	Standard Error
*t*	Student’s *t*-test/regression *t*-statistic
U	Mann–Whitney U test statistic
Wald	Wald test statistic
z	Standardized Z-score (Mann–Whitney/Logistic regression indicator)
chi^2^	Chi-square statistic (Model fit indicator)
β	Standardized regression coefficient
IQR	Interquartile Range
VIF	Variance Inflation Factor
